# Novel management of pseudomonas biofilm-like structure in a post-pneumonectomy empyema

**DOI:** 10.3389/fcimb.2024.1458652

**Published:** 2024-10-17

**Authors:** Alexandra M. Gustafson, Carolina M. Larrain, Lindsay R. Friedman, Rachel Repkorwich, Ifeanyichukwu U. Anidi, Karen M. Forrest, Kevin P. Fennelly, Shamus R. Carr

**Affiliations:** ^1^ National Institutes of Health, National Cancer Institute, Surgery Branch, Bethesda, MD, United States; ^2^ National Institutes of Health, National Cancer Institute, Thoracic Surgery Branch, Bethesda, MD, United States; ^3^ National Institutes of Health, National Heart, Lung and Blood Institute, Critical Care Medicine and Pulmonary Branch, Bethesda, MD, United States; ^4^ Medical Research Council Unit the Gambia, London School of Hygiene and Tropical Medicine, Fajara, Gambia

**Keywords:** biofilm-like structure, pseudomonas, tuberculosis, empyema, dornase, alfa, pneumonectomy

## Abstract

We present a patient with a post-pneumonectomy empyema refractory to surgical debridement and systemic antibiotics. The patient initially presented with a bronchopleural fistula and pneumothorax secondary to tuberculosis (TB) destroyed lung, which required a pneumonectomy with Eloesser flap. Ongoing pleural infection delayed the closure of the Eloesser flap, and thoracoscopic inspection of his chest cavity revealed a green, mucous biofilm-like structure lining the postpneumonectomy pleural cavity. Cultures identified pan-susceptible *Pseudomonas aeruginosa.* Despite debriding this biofilm-like structure and administering systemic antibiotics, the patient continued to show persistent signs of infection and regrowth of the film. We employed a novel approach to dissolve the biofilm-like structure using intrapleural dornase alfa followed by intrapleural antibiotic washes. After 3 weeks of daily washes, repeat inspection demonstrated the biofilm-like structure had completely resolved. Resolving the pseudomonas biofilm-like structure allowed permanent closure of his chest without further need for systemic antibiotics. At follow up 3 months later, he showed no sequalae. This treatment option can be an important adjunct to improve likelihood of chest closure in patients with post-pneumonectomy empyema that resists standard treatment options due to biofilm formation.

## 1 Introduction

Empyema in patients who have undergone pneumonectomies, while uncommon, carries a high mortality. Diagnosing empyema can be challenging due to an insidious onset, but typically presents with fevers, leukocytosis and elevated C-reactive protein (CRP). Cultures of the pleural space most often yield growth of *Pseudomonas aeruginosa* or *Staphylococcus aureus* (Deschamps et al., 2001). Postpneumonectomy empyema outcomes are typically worse in patients with a bronchopleural fistula (BPF). Treatment focuses on debriding the pleural cavity, ensuring adequate drainage, and systemic antibiotics, which have variable penetration to pleural tissue (Teixeira et al., 2000; Bedawi et al., 2023). Despite limited advancements in the management of post-pneumonectomy empyema, standard care still carries elevated morbidity and mortality, with some studies showing a 7.1% operative mortality and 5-year survival of 44.5% (Zaheer et al., 2006).

Treatment options for empyema often require operative intervention, especially when a BPF is present. Historically, treatment involved an open thoracotomy and rib resection, as described by Clagett and Geraci (1963). The current approach, however, involves using video-assisted thoracoscopic surgery (VATS) to debride the pleural cavity and either fill the pleural space with an antibiotic solution or obliterate it by filling the space with various tissue flaps (Azevedo et al., 2020). Despite these new operative techniques, empyema may still be recalcitrant to standard therapy due to growth of a bacterial associated biofilm that may provide a sheltered environment allowing the bacteria to persist despite direct appropriate antibiotic treatment (Yin et al., 2019). Intrapleural administration of dornase alfa (Pulmozyme^®^, Genentech, USA), a DNase, has been used to treat loculated pleural infections (Rahman et al., 2011). In our patient, we used 5 mg of dornase alfa along with intrapleural amikacin to resolve the pleural infection. This novel technique successfully cleared the pleural biofilm-like structure and allowed for closure of the open chest. We present this case, demonstrating the use of intrapleural dornase alfa to treat a bacterial biofilm-like structure contributing to a post-pneumonectomy empyema that was recalcitrant to intrapleural antibiotics. The persistent empyema initially prevented closure of the open thoracotomy window used in the management of this patient.

## 2 Case presentation

A 20-year-old male from The Gambia, West Africa was admitted locally with a right sided hydro-pneumothorax, initially managed with a chest thoracostomy tube. Chest radiographs demonstrated left upper lobe patchy opacities. At that time sputum microscopy was positive for acid-fast bacilli (AFB), and the Xpert MTB/RIF molecular assay of the sputum detected *Mycobacterium tuberculosis* that was negative for resistance to rifampin. However, due to local economic issues and standard of care, sputum cultures were not performed. His serology for HIV was negative.

**Figure 1:**
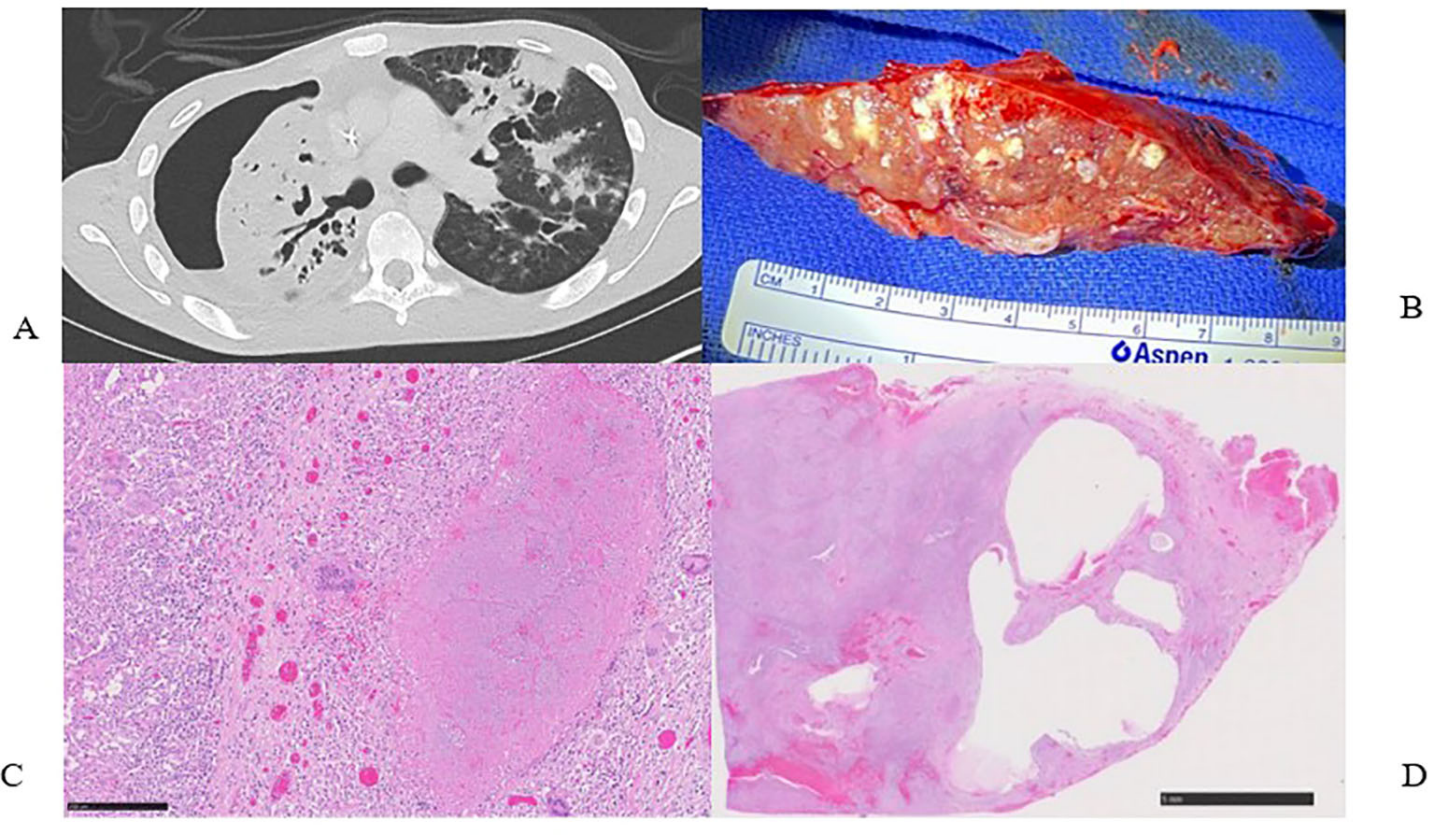
**(A)** Pre-operative computed tomography scan showing right hydropneumothorax, right lung consolidations and collapse. **(B)** Surgical specimen, right pneumonectomy with innumerable micro abscesses and caseating granulomas. **(C)** H&E stain of surgical specimen showing large necrotizing granuloma surrounded by multinucleated giant cells and epithelioid histiocytes (250µm). **(D)** Low power view of lung parenchyma showing diffuse involvement by granulomatous inflammation and cystic lesions (5mm).

The patient began standard daily treatment with rifampin 600 mg, isoniazid 300 mg, pyridoxine 1600 mg, and ethambutol 1100 mg. Despite the chest thoracostomy tube, his lung remained entrapped (**Figure 1A**), and he continued to experience fever and illness. After one month of treatment, the Xpert MTB/RIF panel was negative, but the patient remained febrile after exhausting all local treatment options. Consequently, his treating team referred him for consultation and additional treatment. Upon admission to our institution in the United States, 8 months after his initial presentation, the patient reported persistent low-grade fevers, a dry cough, and an unintentional 9 kg weight loss since his initial admission. He presented as cachectic, weighing 42 kg, but in no acute distress. He had a right chest tube with Heimlich valve in place, decreased breath sounds over his right chest with a pleural rub, and a resting tachycardia of 140 beats per minute. The neurological examination was remarkable for a lower extremity motor and sensory peripheral neuropathy with a right foot drop.

Pleural fluid from his chest tube grew heavy amounts of *Achromobacter* species, *Stenotrophomonas maltophilia*, and *P. aeruginosa*. The initial Mycobacteria Growth Indicator Tube (MGIT) liquid culture of the pleural fluid reported growth of *M. tuberculosis.* This was later amended as a false positive due to the overgrowth of the gram-negative species in the MGIT. However, the Cepheid Xpert MTB/RIF molecular assay detected *M. tuberculosis* that was not resistant to rifampin in the pleural fluid. The isoniazid and ethambutol were discontinued, and the treatment regimen was modified to oral rifampin, moxifloxacin, pyrazinamide, and trimethoprim-sulfamethoxazole, in addition to intravenous amikacin and meropenem to cover all detected microorganisms.

**Figure 2:**
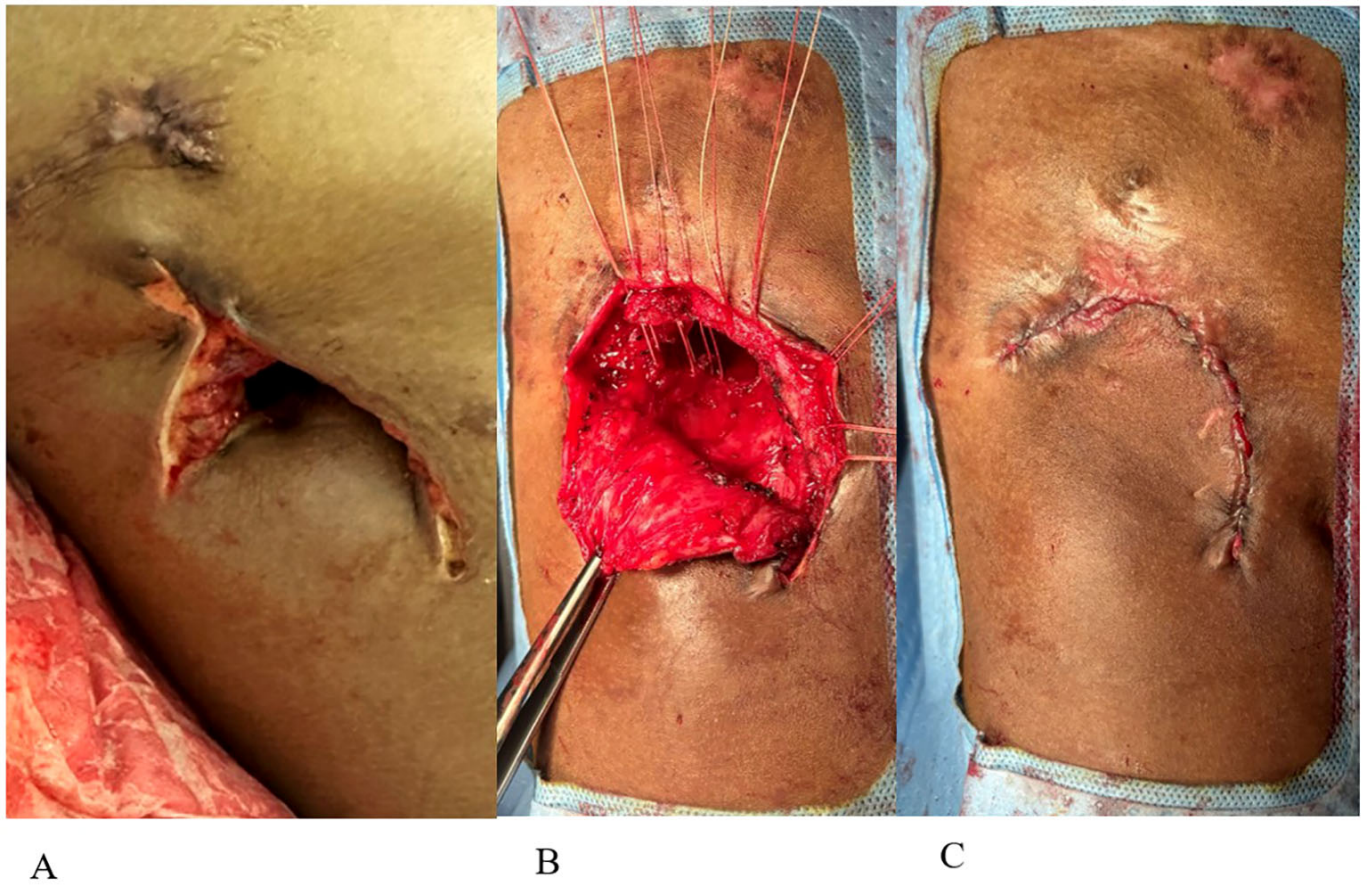
**(A)** Eloesser flap creation. **(B)** Closure of Eloesser flap. **(C)** Final flap closure with skin reapproximated.

The primary team consulted thoracic surgery, and the initial plan aimed to salvage the lung with total pulmonary pleural decortication to permit lung re-expansion to fill the pleural space. However, due to the patient’s malnutrition and poor health status, surgery was delayed until the patient was medically optimized. Over the ensuing 4 weeks, the patient worked with physical therapy and nutrition, eventually regaining 6 kg. The patient underwent a right thoracotomy and pleurectomy with decortication in attempts to preserve the lung. However, inspection of the lung revealed extensive inflammation along with micro abscesses and granulomas involving all three lobes of the right lung (**Figure 1B**). After intraoperative multidisciplinary discussion with pulmonary medicine, the decision was to proceed with a right pneumonectomy. A pedicled rotational pericardial patch was used to buttress the right bronchial stump, and an Eloesser flap was created for open drainage of the infected space (**Figure 2A**).

Final pathology demonstrated necrotizing and non-necrotizing granulomas involving almost the entire right lung parenchyma (**Figures 1C, D**); AFB were present in the necrotizing granulomas. The visceral and parietal pleura was fibrous and contained multiple necrotizing and non-necrotizing granulomas with acid-fast bacilli. All cultures of the resected lung tissue were negative for microbial growth.

**Figure 3:**
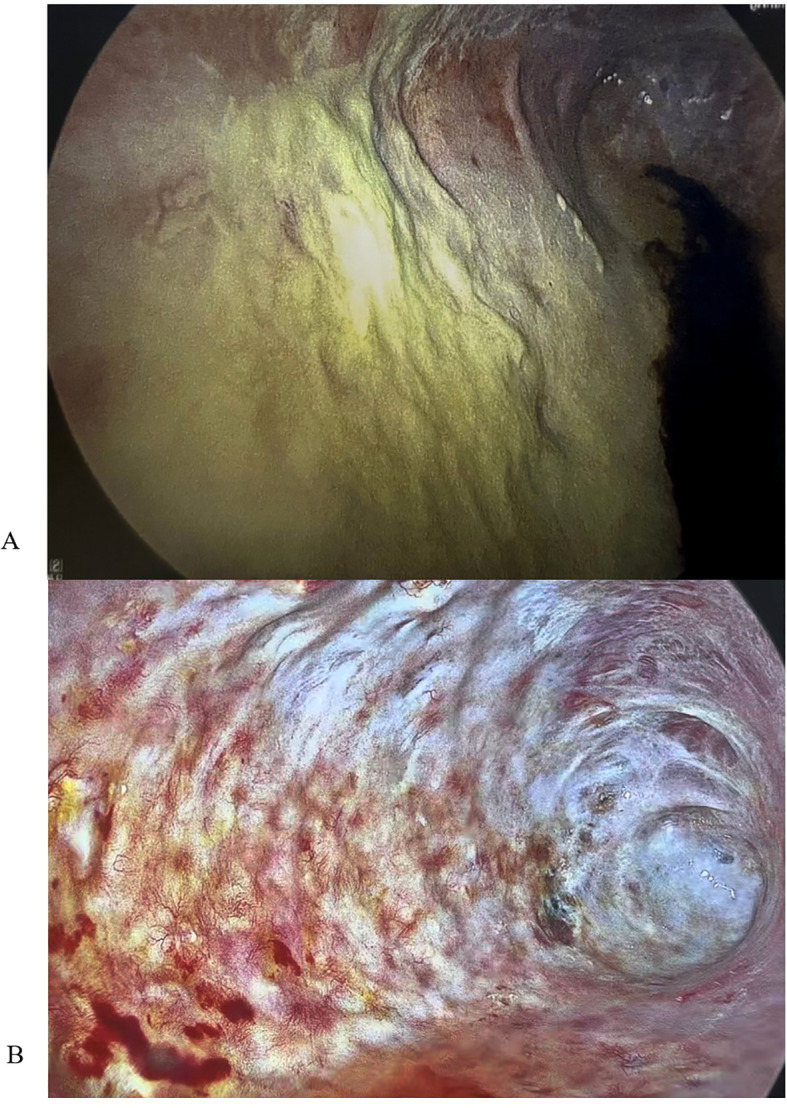
**(A)** VATS image showing biofilm-like structure prior to dornase alfa application. **(B)** VATS image showing resolution of biofilm-like structure after 21 days of dornase alfa application.

The patient experienced an expected Intensive Care Unit stay and transferred to the surgical floor after 11 days. During the immediate postoperative period, he underwent two planned VATS to aid in pleural debridement. There were no signs of gross infection. He continued aggressive management and rehabilitation, and 4 months after his pneumonectomy, plans were formulated to close his Eloesser flap. However, the patient developed a fever with a rising CRP, and leukocytosis. Bronchoscopy with bronchoalveolar washings grew *P. aeruginosa*, and a right sided VATS showed a green film lining the entire right parietal pleura (**Figure 3A**). His chest was debrided and washed out; he received intravenous antibiotics, resulting in eventual improvement. Plans to close the chest were again delayed when a low-grade fever, rising CRP, and leukocytosis recurred. Repeat VATS demonstrated a recurrent green film, suggesting a biofilm-like structure had reformed, with redemonstration of only a pan-sensitive strain of *P. aeruginosa* in pleural microbiology cultures.

After consulting with pulmonology and infectious disease teams, we began intrapleural instillation of 5 mg of dornase alfa in 50 ml of normal saline. The dose of 5 mg was chosen since this has been proven to safely and effectively improve drainage of pleural infections (Rahman et al., 2011). We performed daily dornase alfa instillation followed by intrapleural instillation of antibiotic solution with 500mg amikacin, to which the *P. aeruginosa* was susceptible. The patient then followed a turning protocol to allow the antibiotic solution to contact all areas of the right pleural space. We chose the intrapleural route due to the known poor pleural penetration of systemically administered amnioglycosides (Thys et al., 1988). We monitored serum amikacin levels for up to eight hours after installation due to the unknown systemic absorption of amikacin through an infected pleural space (Thys et al., 1984; Thys et al., 1988).

On the first day of dornase alfa and amikacin instillation, the patient’s serum amikacin level prior to antibiotic administration was <0.8 mcg/mL. We then measured serum amikacin levels 1, 2, 4, and 8 hours post intrapleural administration. The levels were 1.4, 3.7, 3.6, and 1.7 mcg/mL respectively. We repeated this daily; on the subsequent day the patient’s serum amikacin levels were 7.2, 10.4, 9.3, and 3.1 mcg/mL 1, 2, 4, and 8 hours post intrapleural administration. The highest serum amikacin level ever measured was 10.9 mcg/mL. Since maximum safe serum amikacin levels are 20.0.-30.0 mcg/mL (Kovačević et al., 2016), we elected to stop measuring serum amikacin levels while continuing the dornase alfa and amikacin intrapleural washes.

**Figure 4:**
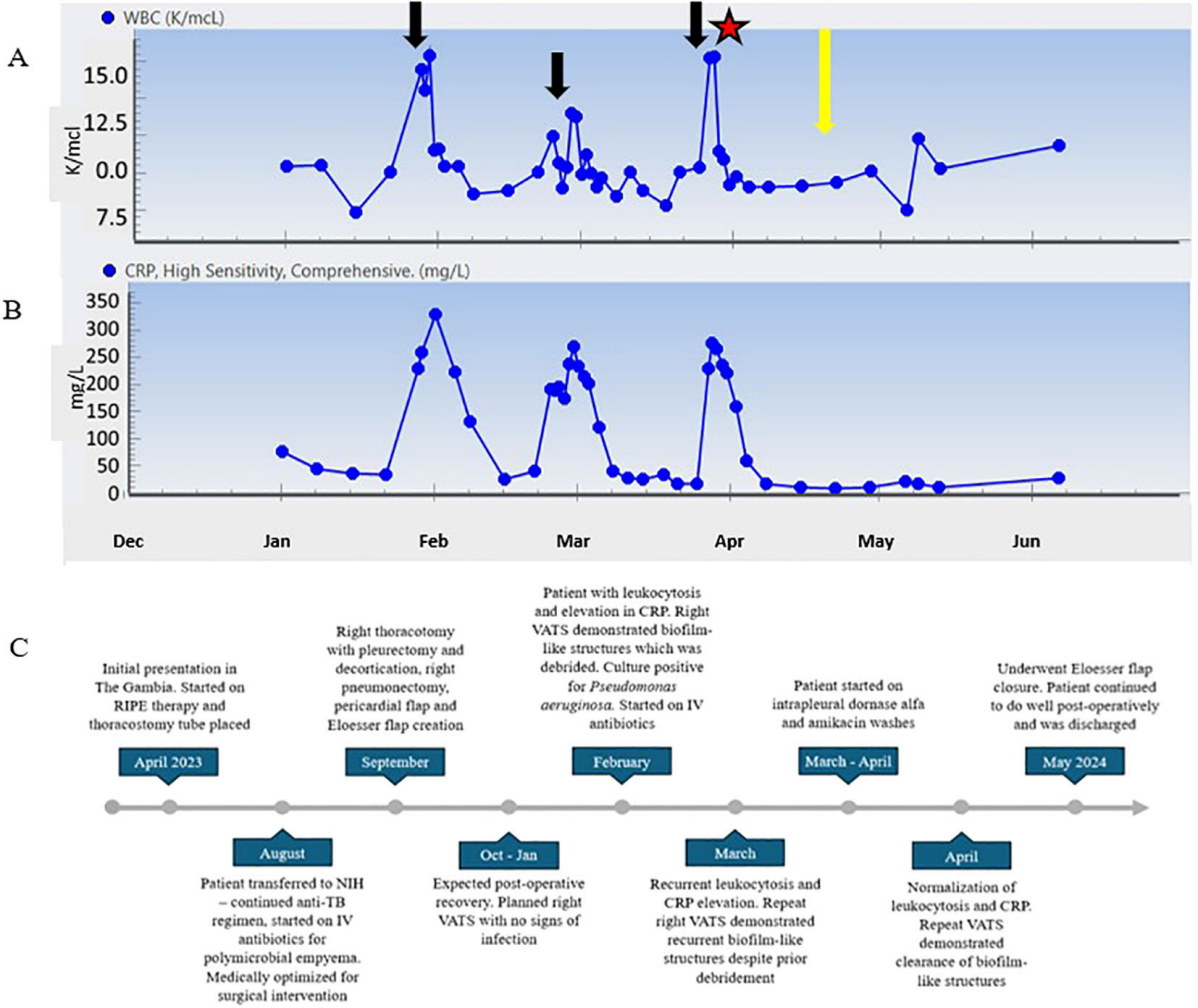
**(A)** WBC count trend throughout clinical course. **(B)** CRP trend from 2 months post operatively until follow up 8 months post operatively. **(C)** Timeline of patient clinical course. Black arrows represent time points at which patient developed symptoms and procedures revealed *P. aeruginosa* growth. Star represents initiation of dornase alfa and amikacin washes. Yellow arrow represents VATS showing resolution of biofilm-like structures.

The patient never exhibited symptoms or signs of amikacin toxicity, and serum levels remained low. Audiometry exams and renal function remained normal. DNase and amikacin washes were repeated daily for 21 days. On subsequent VATS, there was no green film seen, implying resolution of the *P. aeruginosa* biofilm-like structure (**Figure 3B**). Due to no further evidence of infection, we stopped intrapleural dornase alfa administration. Clinically, the patient remained afebrile with no leukocytosis and resolution of the elevated CRP during this time (**Figures 4A, B**). We continued daily amikacin washes only from that time forward, and two weeks later, his Eloesser flap was closed (**Figures 2B, C**).

Immediately prior to closure of the patient’s chest, we instilled 500 mg of amikacin in one liter of normal saline. This solution remained in his chest to fill the pleural cavity as previously described (Zaheer et al., 2006). The patient was discharged home, and on follow up visits he has no complaints and continues to do well (**Figure 4C**).

## 3 Discussion

Post-pneumonectomy empyemas pose a complex clinical challenge. Although occurring in only 5-10% of cases, mortality rates can reach as high as 20% (Schneiter et al., 2001; Zaheer et al., 2006). The hallmark of treatment for post-pneumonectomy empyema involves debridement of infected tissue, pleural drainage, and administration of parenteral antibiotics (Deschamps et al., 2001). Surgeons have developed several techniques to facilitate pleural drainage, the predominant two being Eloesser flap and Clagett window; the main difference is the Eloesser flap is smaller with the possibility of remaining permanent as a drainage site (Denlinger, 2010). While both techniques successfully manage empyemas and result in chest closure in most patients, a small subset are unable to undergo closure. Persistent infection of the pleural space, sometimes associated with a recurrent bronchopleural fistula, often accounts for this. When eradicating bacterial infection from the pleural cavity proves impossible, patients may be left with a permanent drainage window.

Maintaining a chest wall opening, though viable, can impact quality of life. Patients must diligently irrigate the chest cavity and frequently change dressings. The patient we present here experienced recurrent empyema, which repeatedly prevented closure of his chest. For a young, otherwise healthy patient, discharge home with an open chest is less than ideal, particularly if returning to a community with limited health resources. Thus, to treat this recalcitrant empyema, we employed a novel technique. While clinicians frequently instill dornase alfa through a tube thoracostomy to break apart loculated pleural effusions (Thomas et al., 2015), its instillation to destabilize a post-pneumonectomy biofilm, followed by subsequent antibiotic solution, has not been previously described in humans. Although the purpose of dornase alfa in treating infected pleural collections is to reduce the viscosity of recruited leukocytes via hydrolysis of extracellular DNA from exuberant neutrophil extracellular trap formation, we specifically administered dornase alfa to target the bacterial biofilm lining the pleural cavity of the pneumonectomy space. Dornase alfa acts to destabilize the biofilm and permit antibiotics to penetrate the chronically infected pleural space, thereby aiding in eradicating *P. aeruginosa*. Here, we present the successful use of dornase alfa to disintegrate a bacterial biofilm-like structure. We hypothesize the biofilm-like structure contributed to the difficulty in eradication of a polymicrobial post-pneumonectomy empyema preventing closure of an Eloesser flap.

Various microorganisms produce biofilms that share common traits. They often harbor dormant populations of pathogens that cause recalcitrant infections, as seen in our patient with persistent *P. aeruginosa* growth. The extracellular matrix constitutes a major component of biofilms. These unique extracellular matrix compositions contribute to the virulence of biofilms. Moreover, even if antibiotics are bactericidal, the biofilm often remains, sheltering bacteria from the antibiotic and increasing the likelihood of recurrent infections (Koo et al., 2017; Yin et al., 2022). Current strategies to eradicate biofilms must target multiple aspects that lead to biofilm formation. Hence, we employed a multimodal approach with surgical debridement, dornase alfa for mechanical disruption of the extracellular matrix of the biofilm, and pleural instillation of amikacin solution to reach a high concentration of antibiotic directly in the infected tissue. When delivering antibiotics in this manner, there must still be consideration for systemic absorption. Measurements of systemic amikacin levels after pleural wash demonstrated low systemic absorption. This finding differs from other studies using intrapleural amikacin, however these studies were done in a noninfected pleural space (Clagett and Geraci, 1963; Zaheer et al., 2006).

*P. aeruginosa* creates biofilms, a bacterial attribute that leads to chronic infections due to the inability of the antibiotic to penetrate the biofilm. The biofilm matrix primarily comprises proteins, carbohydrates, and DNA; pseudomonal biofilms contain a large amount of extracellular DNA (Allesen-Holm et al., 2006). DNases target and break down these foreign DNA particles by an enzymatic process. Degrading extracellular DNA has many medical applications, one of which is to prevent pulmonary infections (Lauková et al., 2020). In one study, mice deficient in DNase exhibited increased susceptibility to bloodstream infections (Lacey et al., 2023). Additionally, these mice had bacterial lesions that resembled a biofilm and contained bacterial DNA while excluding neutrophils (Lacey et al., 2023). *P. aeruginosa* can cause severe, life-threatening pulmonary infections in patients with cystic fibrosis (CF), and one preventive method involves CF patients inhaling dornase alfa. This acts by breaking up the thick, viscous secretions while also cleaving the extracellular DNA that allows *P. aeruginosa* to form biofilms (Gnanadhas et al., 2015). A study evaluating the use of L-methionine to increase DNase activity showed that chronic infections with *P. aeruginosa* biofilm can be treated with DNase and antibiotics (Gnanadhas et al., 2015). Thus, we extrapolate that the same treatment could be used in patients with recurrent chronic empyemas due to *P. aeruginosa* biofilm, as in our presented patient.

While limitations exist, this report is, to our knowledge, the first to utilize intrapleural dornase alfa and amikacin to dissolve and treat a biofilm-like structure of the pleural cavity. Previously, Deng and colleagues have shown that DNase effectively inhibits early biofilm formation (Deng et al., 2022). However, no *in-vitro* studies have assessed the ability of DNase to treat mature biofilms. Moving forward, this method could be improved by measuring clearance of the biofilm using laboratory testing rather than imaging alone.

## Data Availability

The original contributions presented in the study are included in the article/**Supplementary Material**, further inquiries can be directed to the corresponding author.
