# Supplementary material for: Novel management of pseudomonas biofilm-like structure in a post-pneumonectomy empyema
Source: Front Cell Infect Microbiol. 2024 Oct 17;14:1458652. doi: 10.3389/fcimb.2024.1458652 (PMC11525003; doi:10.3389/fcimb.2024.1458652)
Supplement: Supplementary file 1 [file DataSheet1.docx]

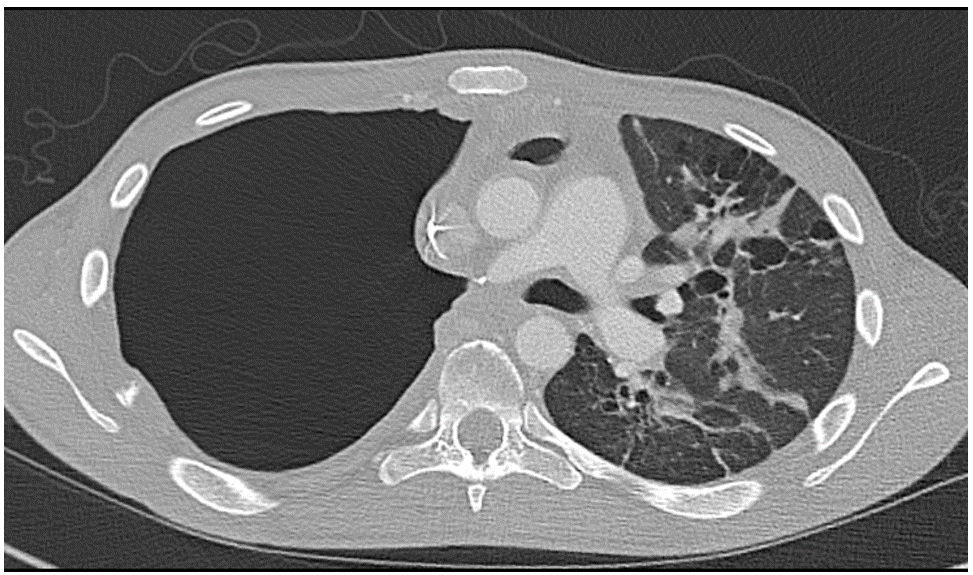


**Supplemental Figure 1: Computed tomography scan of chest two months post pneumonectomy. Patient doing well with on signs of active infection.**


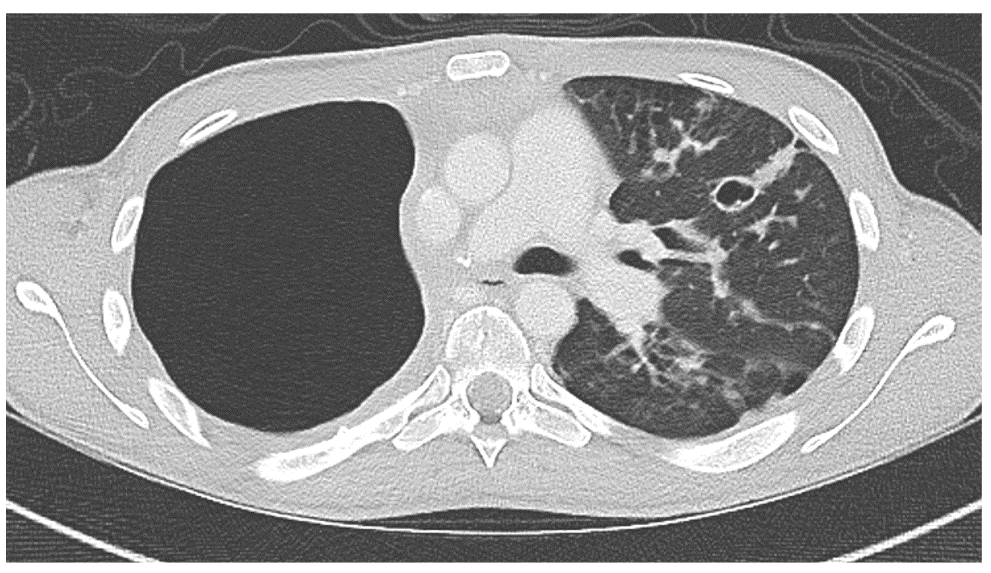


**Supplemental Figure 2: Computed tomography scan of chest 7 months post pneumonectomy. Video assisted thoracoscopic inspection revealed biofilm-like structure similar to figure 3A.**


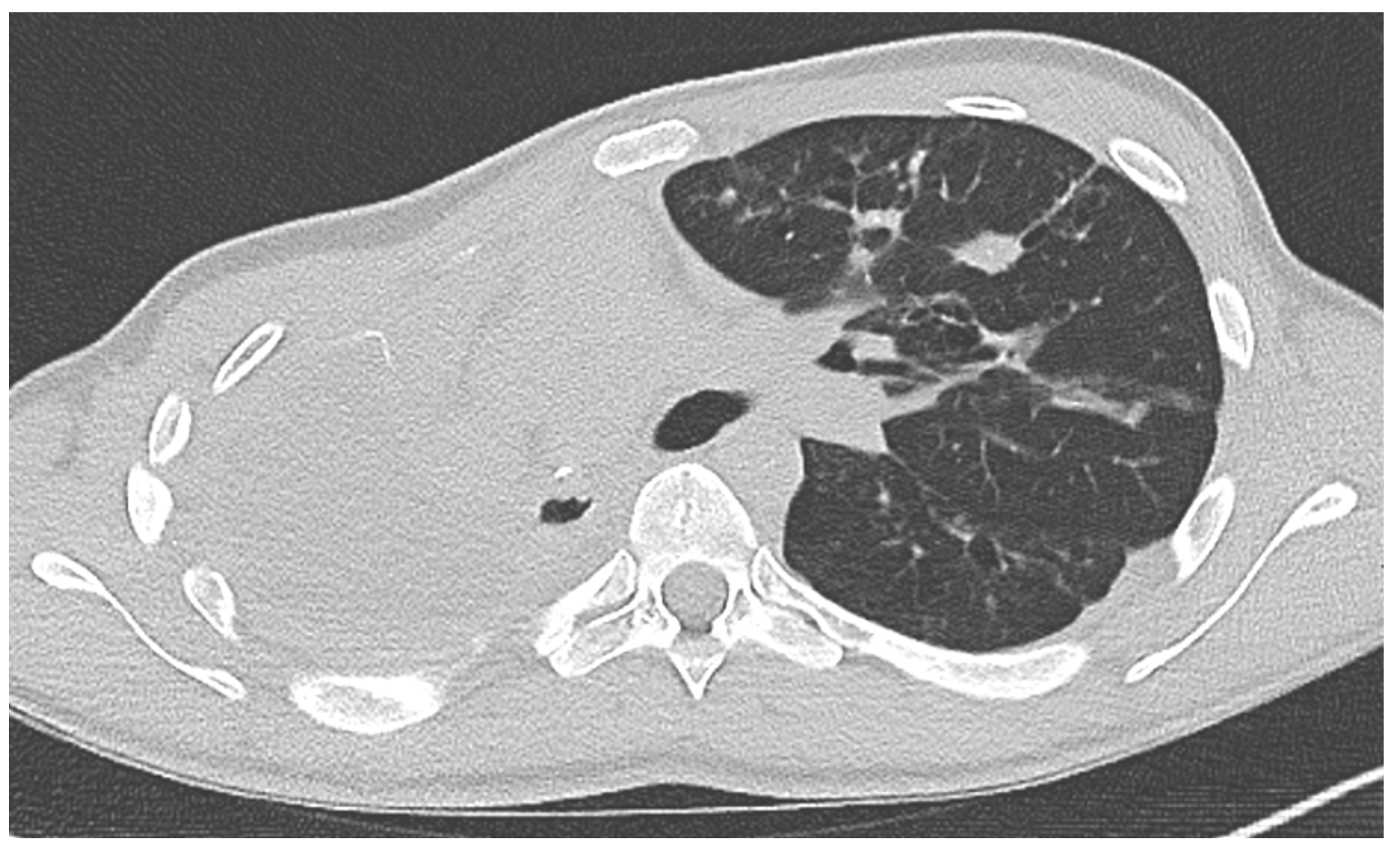


**Supplemental Figure 3: Computed tomography scan of chest after discharge (11 months post pneumonectomy) demonstrating closure of Eloesser flap and obliteration of right pleural cavity with antibiotic solution.**
